# Efficacy of PEGylated ciliary neurotrophic factor superagonist variant in diet-induced obesity mice

**DOI:** 10.1371/journal.pone.0265749

**Published:** 2022-03-22

**Authors:** Maria Rosaria Battista, Antonella Grigoletto, Tommaso Tedeschini, Antonella Cellucci, Fabrizio Colaceci, Ralph Laufer, Gianfranco Pasut, Annalise Di Marco

**Affiliations:** 1 IRBM SpA, Pomezia, Rome, Italy; 2 Department of Pharmaceutical and Pharmacological Sciences, University of Padova, Padova, Italy; 3 Lysogene, Neuilly-sur-Seine, France; Medical University of Vienna, AUSTRIA

## Abstract

Ciliary neurotrophic factor (CNTF) is a neurotrophic cytokine able to induce appetite reduction, weight loss and antidiabetic effects. However, its susceptibility to neutralizing anti-CNTF antibodies in patients hampered its use for treatment of human obesity and diabetes. In addition, CNTF has a very short plasma half-life, which limits its use as a therapeutic agent. Solutions, directed to prolong its *in vivo* effects, vary from the implantation of encapsulated secreting cells to identification of more active variants or chemical modification of the protein itself. PEGylation is a widely used modification for shielding proteins from circulating antibodies and for increasing their plasma half-life. Here, we have selected DH-CNTF, a CNTF variant which has a 40-fold higher affinity for the CNTF receptor α accompanied by an increased activity in cellular assays. The PEGylated DH-CNTF retained the biological activity of native protein *in vitro* and showed a significant improvement of pharmacokinetic parameters. In an acute model of glucose tolerance, the PEG-DH-CNTF was able to reduce the glycemia in diet-induced obese animals, with a performance equaled by a 10-fold higher dose of DH-CNTF. In addition, the PEGylated DH-CNTF analog demonstrated a more potent weight loss effect than the unmodified protein, opening to the use of CNTF as weight reducing agent with treatment regimens that can better meet patient compliance thanks to reduced dosing schedules.

## Introduction

Ciliary neurotrophic factor (CNTF), originally described as a trophic factor for chick ciliary ganglion neurons *in vitro* [1], was later demonstrated to belong to the gp130 cytokine family along with interleukin-6, interleukin-11, Cardiotrophin-1 (CT-1), Oncostatin M (OSM), and Leptin [2]. CNTF is expressed in glial cells, in peripheral nerves and in the CNS and it has been shown to enhance the survival and differentiation of neurons in the central and peripheral nervous system [3] and is thought to elicit its cytoprotective effects through activated release after stress or injury. CNTF also has non-neuronal effects including initiation of an acute phase response in liver cells, as well as producing a myotrophic effect on denervated skeletal muscles [4]. CNTF synthesis in the CNS is increased in trauma, sepsis, and cancer, all of which are associated with anorexia [5]. CNTF elicits its actions through a receptor complex consisting of the ligand-specific α-receptor CNTFRα, and β-receptors glycoprotein 130 (gp130) and leukemia inhibitory factor receptor β (LIFRβ) [6]. Lacking transmembrane and cytoplasmic domains, CNTFRα is anchored to the cell membrane by a glycosylphosphatidylinositol linker; cleavage of this linker can release soluble receptor, sCNTFRα, rendering cells expressing just gp130 and LIFRβ also capable of responding to CNTF [7].

The CNTF pathway has been proposed as a target for the treatment of obesity associated with leptin resistance. Systemic administration of CNTF activated genes in the arcuate nucleus, the hypothalamic region of the brain involved in the regulation of energy balance [8, 9]. Moreover, administration of CNTF in leptin-resistant models of obesity, namely ob/ob, db/db, and high-fat–fed (diet-induced obesity) mice, was found to result in hypophagia, reduced body weight, and attenuated hyperinsulinemia.

Axokine is a modified version of human CNTF in which the C-terminus has been truncated by 15 residues and, to enhance the in vitro potency and stability of the molecule, glutamine was replaced by arginine at position 63 and the free cysteine at position 17 replaced by alanine [10]. This analog of CNTF has up to 5 times greater potency than CNTF. Axokine was evaluated in phase III trials involving 1467 obese subjects treated with subcutaneous injections of Axokine, compared to 501 placebo treated subjects for one year. This study demonstrated that Axokine-treated patients lost 5% of their initial body weight compared to placebo-treated patients. However, despite initial success in patients, the drug was discontinued because most patients treated with Axokine developed anti-drug (ADA) antibodies. Thus, overcoming the immunogenicity of Axokine would be an important step to improve efficacy and durability of its effects.

CNTF has a short plasma half-life [11], which limits its use as a therapeutic agent. Chemical modifications of proteins such as PEGylation is one successful strategy to increase plasma half-life and to reduce immunogenicity [12, 13]. The known drawback of losses in target affinity after polymer conjugation can be minimized by site-directed PEGylation, linking the PEG polymer far away from the receptor binding sites. During the years, different chemical and enzymatic methods of PEGylations have been devised, allowing the coupling of PEG at selected sites such as a cysteine residue, the *N*- and C-terminus, a disulfide bond, a glutamine via transglutaminase, a glycan via sialyltransferase, etc [14–18].

Previous PEG20K modifications and transferrin (Tf) coupling based on the natural free thiol of Cys17 residue of the wild-type CNTF molecule, have been reported [19]. Both conjugates led to weight-loss in mice, with PEG20k-CNTF being more potent than Tf-PEG5k-CNTF. However, the weight-reducing effect of CNTF was evaluated in normal mice and not in a model of diet-induced obesity (DIO), which resembles most of the key features of the human metabolic syndrome.

In the present study, the effect of site-specific PEGylation was examined in the background of DH-CNTF variant (S166D/Q167H), which was previously shown to have greatly enhanced CNTFRα-binding affinity [20]. This analog preserved its biological activity after PEGylation at Cys17, with an important gain in stability. The PEGylated DH-CNTF was able to induce weight loss in DIO mice accompanied by a reduction in hyperglycemia and insulin normalization.

The greater biological activity and stability of PEGylated DH-CNTF could allow the use *in vivo* of lower doses and reduced dosing frequency, together with a decrease of the immunogenicity potential.

## Materials and methods

### Production of recombinant DH-CNTF

Recombinant human DH-CNTF (human CNTF harboring two protein mutations S166D and Q167H in the D helix) and wild-type human CNTF were prepared and purified as previously described [21]. Additional purification was carried out by RP-HPLC using a C18 column (Vydac 218TP1010; 25 × 1 cm; 10 μm). DH-CNTF was eluted at a flow rate of 3 mL/min, with a gradient of water and acetonitrile, both containing TFA 0.1% (v/v) (gradient: 0’-5% B, 5’-40% B, 18’-66% B, 20’-90% B, 23’-5% B), monitoring absorbance at 280 nm. Prior to lyophilization for removal of the solvent, n-octylglucopyranoside was added at 0.1% (w/v). The purified DH-CNTF was then reconstituted in demineralized water for storage at 4°C before use. Recombinant proteins were analyzed on reducing and non-reducing SDS/PAGE gels stained with Coomassie blue. They contained less than 5 ng of endotoxin per mg of protein as determined by the *Limulus* amoebocyte assay (Merk, Darmstadt, Germany). Bradford reagent was used for measuring protein concentration.

### PEGylation of DH-CNTF

To the purified DH-CNTF solution (protein conc. 0.5–1 mg/mL) urea was added to the final concentration of 4 M. Then, 5 equivalent of PEG-Mal (20 kDa) were added and the reaction was let to proceed for 4 h at room temperature. The reaction was monitored by RP-HPLC, indicating that about 60% of the protein had reacted after 2 h and a maximum of 63% was reached at 4h. The reaction mixture was purified by RP-HPLC as described above (gradient: 0’-5%, 5’-40%, 22’-53.6%, 25’-90%, 28’-5% B), monitoring absorbance at 280 nm. The fractions corresponding to PEG-DH-CNTF were collected, pooled, supplemented with n-octylglucopyranoside (0.1% w/v), lyophilized and finally reconstituted in demineralized water.

### Characterization of PEG-DH-CNTF

DH-CNTF and PEG-DH-CNTF were characterized by RP-HPLC, SDS-PAGE, circular dichroism and mass spectrometry.

#### RP-HPLC analysis

Samples were loaded onto a C18 column (Phenomenex Jupiter 250 × 4.6 mm; 5 μm; 300 Å) and elution was performed at a constant flow of 1 mL/min., with a gradient of water and acetonitrile, both containing TFA 0.1% (v/v), gradient: 0’-5%, 5’-40%, 25’-80%, 27’-90%, 30’-5% B, recording signal at 280 nm. Typically, DH-CNTF eluted at about 17.8 minutes and PEG-DH-CNTF at about 16.9 minutes (S1 File).

#### SDS-PAGE

Electrophoresis (SDS-PAGE) was performed in accordance with the Laemmli-SDS-PAGE protocol [22]; Electrophoretic runs were made using an Electrophoresis Power Supply 300 (Pharmacia, New Jersey, USA). The gel was firstly stained with iodine for PEG detection and then with Coomassie Blue for protein detection as reported elsewhere [18].

#### FUV-CD

Far-UV circular dichroism spectra were measured on a Jasco J-810 spectropolarimeter equipped with a Peltier temperature control unit at 25°C. The samples were dissolved in PBS pH 7.4 at a protein concentration of 0.1–0.2 mg/mL. The spectra were collected between 200 and 250 nm with an average of 3 scans and the data at each wavelength were averaged for 8 s. The sample cell path length was 1 mm. The CD data were converted to mean residue ellipticity, expressed in deg cm^2^ dmol^−1^ by applying the following formula:

$$\Theta =\Theta_{obs}\;(MRW)/10L[C]$$

where ϴ is the observed ellipticity in degrees, the MRW is the mean residue weight of the protein (molecular weight divided by the number of amino acids), [C] is the protein concentration in mg/mL, and L is the optical path length in centimeters. Spectra were recorded at 25°C, at 95°C and after melting in samples heated at 95°C and cooled back to 25°C as elsewhere reported [23].

#### MALDI-TOF MS

Mass spectra were obtained with a REFLEX time-of-flight instrument (4800 Plus MALDI TOF/TOF, AB Sciex, Framingham, Massachusetts, USA) equipped with a SCOUT ion source, operating in positive linear mode. A pulsed UV laser beam (nitrogen laser, λ 337 nm) generated ions that were accelerated to 25 kV. Matrix (a saturated solution of sinapic acid in water/ACN (1:1, v/v) + 0.1% TFA (v/v)) was mixed with an equal volume of sample, and 1 μL was loaded on the plate.

### Determination of the site of PEG conjugation

DH-CNTF and PEG-DH-CNTF (15 μg) were analysed by SDS-PAGE. The respective slices were isolated and washed for three times with 200 μL of a 0.1 M NH_4_HCO_3_/CH_3_CN 50:50 (v/v) mixture at pH 7.8 for 10 min. After dehydration with CH_3_CN for 15 min, gel slices were completely dried in a speed-vac system. Native and conjugated protein were incubated with 200 μL of 5 mM tris(2-carboxyethyl)phosphine (TCEP) in 0.1 M NH_4_HCO_3_ pH 7.8 for 10 min at 60°C to reduce the disulfide bridges and alkylated by adding 200 μL of 55 mM iodoacetamide (IAA) in 0.1 M NH_4_HCO_3_ pH 7.8 for 15 min at 37°C in the dark. Gel slices were washed twice with a 0.1 M NH_4_HCO_3_/CH_3_CN 50:50 (v/v) mixture at pH 7.8 for 10 min, dehydrated with CH_3_CN for 15 min and again completely dried. 50 μL of trypsin protease (Thermo Fisher Scientific; 10 μg/mL in 0.1 M NH_4_HCO_3_ pH 7.8) were added and the digestion reaction was let to proceed overnight at 37°C and 300 rpm in a thermomixer. Formic acid to a final concentration of 2.5% (v/v) was added and the peptides were extracted for three times with 100 μL of a H_2_O/CH_3_CN (+ TFA 0.1%) 50:50 (v/v) mixture. After concentration, the digested mixtures were analysed using an UPLC-QTOF system. The ACQUITY UPLC H-Class (Waters) was equipped with an AdvanceBio Peptide Map Guard (2.1 × 5 mm, 2.7 μm; Agilent) and AdvanceBio Peptide Mapping column (150 × 2.1 mm, 2.7 μm; Agilent), maintained at 30°C and a flow rate of 0.2 mL/min, and eluted with a solvent gradient of water/CH_3_CN both containing 0.1% formic acid. Gradient 2′–2% ACN, 38′–65% ACN, 40′–98% ACN, 43′–98% ACN, 44′–2%. The Xevo G2-S QTof (Waters) was operated in the ESI positive ion, resolution mode, with a detection window between 50–2000 m/z. Analysis were performed at a capillary voltage of 1.5 kV, at a cone voltage of 40.0 V and source offset of 80 V. MS^E^ acquisition was performed by alternating two MS data functions: one for the acquisition of the peptide mass spectra with the collision cell at low energy (6 eV) and the other for the collection of the peptide fragmentation spectra with the collision cell at elevated energy (linear ramp 20 to 40 eV). Analyses were performed with LockSpray™ using a solution of 1 ng/μL LeuEnk MS in 50: 50 (v/v) water/CH_3_CN containing 0.1% formic acid, sampled every 45 s. MS^E^ data were processed with MassLynx and BiopharmaLynx 1.3.4 Software (Waters). Trypsin was set up as the digest reagent and 2 missed cleavages were allowed. The MS ion intensity threshold was set at 250 counts, and the MS^E^ threshold was set to 100 counts. Both MS mass match tolerance and MS^E^ mass match tolerance were set to 15 ppm.

### *In vitro* biological assay: Haptoglobin secretion in the hepatoma cell line HepG2

Induction of haptoglobin synthesis and secretion from HepG2 cells was determined as previously described [24] with minor modifications. Briefly, HepG2 cells were plated in 96-well plates at the density of 50,000 cells/well and left to reach 80% confluency. Confluent monolayers were washed with assay medium (serum-free culture medium containing 1 μM dexamethasone) and then treated for 24 hours with CNTF analogs, in the presence of 10 ng/mL s-CNTFRα. The amount of haptoglobin secreted into the culture medium was determined using a commercial ELISA (GenWay Biotech), EC_50_ was calculated from dose-response curves by nonlinear regression using XLfit 4.2 (IDBS Ltd), applying the 4 parameter logistic model. The haptoglobin secretion assay was also used for the quantification of plasma concentrations of biologically active analogs from *in vivo* studies.

### Animal studies

Mice and rats were obtained from Charles River S.p.A. Calco (Como) Italy. A pre-dose acclimatization period of 5 days was allowed, during which time the health status of the animals was assessed. Before and during the tests, the animals were housed in Individual Ventilated Cages (IVCs Tecniplast) with sawdust as bedding (three animals per cage). Cages were identified by a color code label showing the sample ID, animal number and details of treatment (route, dose and sex). Animals were identified by a unique number on their tail via permanent marker. Animal room controls were set to maintain the temperature within the range of 20 to 24°C and relative humidity within the range of 40 to 70% and an average daily airflow of at least 10 fresh air changes per hour. Actual conditions were recorded. The room was lit by controlled fluorescent tubes to give an artificial 12-hour light/12-hour dark cycle each day. Animals were offered drinking water supplied to each cage via a water bottle and a commercially available laboratory rodent irradiated diet (Mucedola 4RF21, Mucedola S.r.L. Settimo Milanese, Milano-Italy) *ad libitum*.

### Pharmacokinetic studies

For pharmacokinetic studies, animals were fasted overnight before dosing and housed individually. Studies were conducted in groups of female 6- to 14-week old NMRI mice, and male 7-week old Wistar Han rats. Blood sampling was performed using retro-orbital bleed in mice, while for rats, the fully automated system DiLab^®^ AccuSampler^®^ was used, through a surgically implanted catheter in the carotid artery. Blood was collected into heparinized tubes, then plasma was separated by centrifugation and samples were kept frozen (-20°C), until assayed. All surgeries were carried out under inhalation of the anesthetic agent isoflurane (2%), using the anaesthesia system Vet-Tech (Vet-Tech, UK),

Animals received an intravenous dose of 0.3 mg/kg CNTF or PEG-DH-CNTF dissolved at 2 mL/kg in 0.9% saline containing 0.2 mg/mL endotoxin-free bovine serum albumin, via the tail vein. *In vivo* IV and SC PK studies required 12 and 15 mice per study, respectively: three terminal blood samples per compound per each time points (IV.: pre-dose; 0.08 h; 0.17 h; 0.25 h; 0.50 h; 1 h and 4 h and SC.: pre-dose; 0.08 h; 0.25 h; 0.50 h; 1 h; 2 h; 4 h; 6 h; 8 h, 24 h; 48 h and 72 h). Blood sampling was performed as indicated in S1 File.

Mice were dosed subcutaneously with 1 mg/kg, dissolved at 5 mL/kg. Pharmacokinetic parameters were calculated by non-compartmental analysis using WinNonlin (Pharsight). Plasma clearance (CLp) was calculated as the intravenous dose divided by the area under the plasma concentration–time curve (AUC) from zero to infinity. The apparent half-life (T_1/2_) was estimated from the slope of the terminal phase of the log plasma concentration–time curve. Bioavailability (F) was estimated as the AUC ratio following oral and IV administration normalized for differences in dose.

### Weight loss

Experiments were performed using groups of male C57BL/6J Diet-induced Obesity (DIO), starting at 23 weeks of age. Male DIO mice were used for weight loss to compare with previous studies from our group [8]. One week before the start of the study, animals were housed in individual cages with *ad libitum* access to water and 60% fat diet, under a12-h light-dark cycle. Animals were accustomed to daily intraperitoneal injections of vehicle (0.9% saline/0.2mg/mL endotoxin-free bovine serum albumin for two days before the beginning of the daily subcutaneous treatment (day 1) with either vehicle or CNTF analogs for 5 days. Animals were weighted after injection and food intake was determined by recording the amount of chow remaining in the food dishes.

### Glucose tolerance test *in vivo*

Male diet-induced obese (DIO) mice were accustomed to daily subcutaneous injection with vehicle (0.9% saline with 0.2 mg/mL endotoxin-free bovine serum albumin thereafter named vehicle) for 2 days before experiment. Weight values were monitored accordingly, and groups were formed based on equal body weight the day before experiment. Animals (6 mice/group) were treated with an intraperitoneal glucose solution of 0.3 mg/mL (1.5 g/kg), 6 hours after the subcutaneous injection of DH-CNTF (1 mg/kg), PEG-DH-CNTF (0.2 mg/kg) and vehicle. Blood samples were collected from the tail vein 30 minutes before intraperitoneal glucose dosing and every 30 minutes after until the last sampling at 120 minutes. Glucose was measured with Breeze 2 (ASCENSIA_®_ Blood Glucose Monitoring System). The means of AUC and C_max_ were compared using one-way analysis of variance (ANOVA, post-hoc Dunnett’s Multiple Comparison Test).

### Ethics statement

The study protocol was approved by the Ethics Committee of the IRBM SpA and the Italian Ministry of Health. The animals were handled in compliance with Italian Legislative Decree 116/92 guidelines and in accordance with “The Guide for the Care and Use of Laboratory Animals” published by the National Research Council of the National Academies. This study was conducted in full compliance with the EU Directive 63/2010 (On the Protection of Animals used for Scientific Purposes) and its Italian transposition (Italian Decree no. 26/2014) as well as with all applicable Italian legislation and guidelines. In particular, this project was submitted for comments and approval to the internal IRBM IACUC and then submitted to the Italian Ministry of Health for government authorization. IRBM is authorized by the Italian Ministry of Health and by local veterinary authority to house, breed and use laboratory rodents for scientific purposes. All surgical procedures were performed under isoflurane anesthesia. All surgical procedures were performed under isoflurane anesthesia and animals were euthanized by carbon dioxide inhalation, with all efforts made to minimize suffering. Animals were euthanized by carbon dioxide inhalation followed by cervical dislocation, with all efforts made to minimize suffering.

### Statistics

Data were expressed as the means ± SE. Statistical significance was assessed by ANOVA with conventional P < 0.05 value. All statistical analyses were performed using GraphPad Prism (version 8.4.2)

## Results and discussion

### Biological activity of DH-CNTF compared with the wild-type CNTF

DH-CNTF was previously identified as a superagonist [20] based on its 40-fold higher affinity for the CNTFRα, the sub-unit which confers CNTF responsiveness to the signaling complex formed by gp130 and LIFR. It was chosen for PEGylation because a lower dose of the unmodified DH-CNTF was able to achieve the same biological effect as the wild-type protein upon subcutaneous administration in animal models of obesity [8]. The evaluation of the biological activity confirmed the greater potency of DH-CNTF, as indicated from the quantification of haptoglobin secretion in HepG2 cells, in the presence and in the absence of different concentrations of soluble CNTFRα.

The cell line was used as a cellular assay system to confirm the intrinsic biological activity (i.e. potency to activate CNTF receptors) of the recombinant proteins, produced in bacteria, before in vivo studies.

The human hepatoma cell line HepG2 does not express CNTFRα and responds only to high concentrations of the cytokine, but in the presence of exogenous s-CNTFRα, cells become sensitive to low concentrations of CNTF, due to the formation of the high-affinity tripartite receptor complex [21].

**Fig 1:**
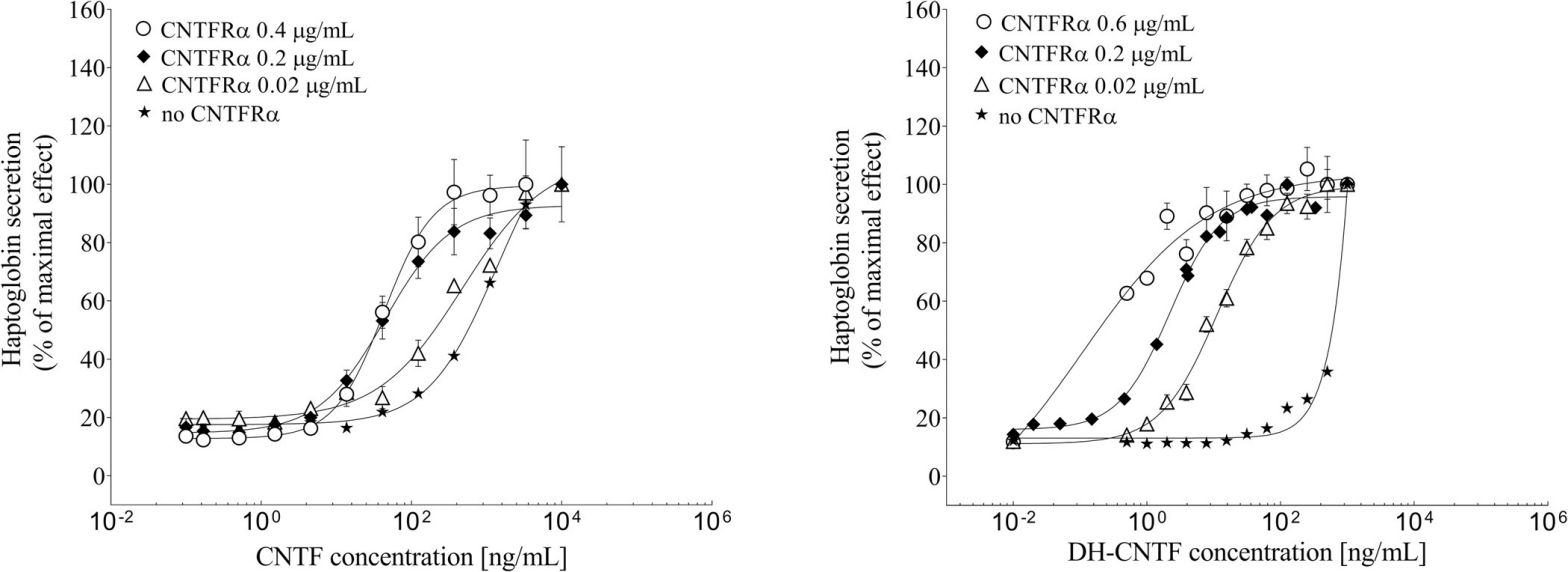
Biological activity of CNTF and DH-CNTF. HepG2 cells were treated with CNTF and DH-CNTF in the absence and presence of different amount of soluble CNTFRα. Results were expressed as a percentage of the maximal CNTF-induced response. Each concentration point was the mean ± S.E. from at least three independent experiments.

As reported in Fig 1 and Table 1, DH-CNTF was about 30-fold more potent than CNTF in this assay. Addition of increasing concentrations of soluble CNTFRα resulted in a dose-dependent reduction of the EC_50_ for both CNTF and DH-CNTF, because of the formation of high affinity CNTF receptor complexes [24]. DH-CNTF reached a limiting EC_50_, reflecting cytokine saturation with the soluble receptor, but at lower concentration of receptor than CNTF, due to its greater affinity for the CNTFRα.

**Table 1:** Potency (EC_50_, mean ± S.E.) of CNTF and DH-CNTF in the absence and in the presence of different amounts of soluble CNTFRα.

	CNTFRα [μg/mL]	EC_50_ [ng/mL]
**CNTF**	0.4	43 ± 2
	0.2	63 ± 9
	0.02	320 ± 44
	No Receptor	717 ± 54
**DH-CNTF**	0.6	0.4 ± 0.1
	0.2	2.3 ± 0.2
	0.02	12 ± 1
	No Receptor	577 ± 52

### PEGylation of DH-CNTF

**Fig 2:**
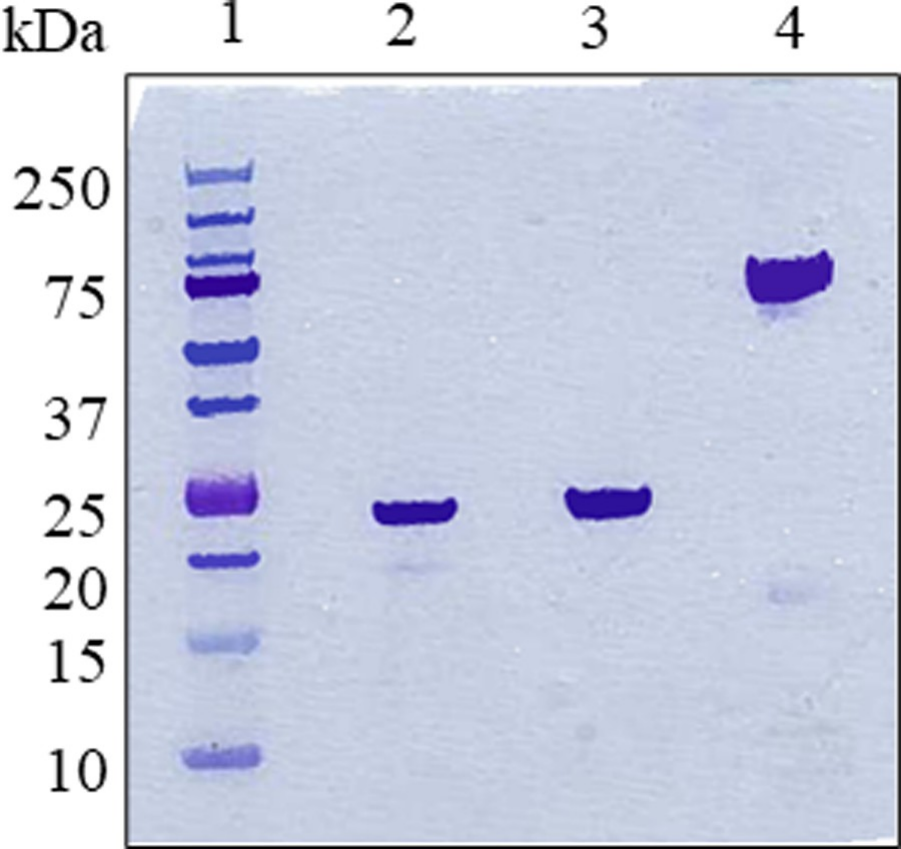
SDS-PAGE analysis. MW markers (lane 1), purified starting material (DH-CNTF, lanes 2 and 3) and PEG-DH-CNTF (lanes 4).

As a first step in the design of conjugated DH-CNTF variants, it is important to identify regions and specific residues of the cytokine where introduction of a PEG moiety will not lead to loss of biological activity. CNTF interacts with 3 different receptor subunits, CNTFRα, gp130 and LIFR. The structure of CNTF has been solved by X-ray crystallography [25] and a variety of mutagenesis studies have allowed to roughly define the sites of interaction of CNTF with its individual receptor subunits. In analogy with other gp130 cytokines, LIF and IL-6, CNTF has a three-dimensional fold, the four-helix bundle [26], which comprises four helices (termed A, B, C and D) linked by polypeptide loops. The binding site for CNTFRα (site 1) involves residues on the C-terminal part of helix D and loop A/B. The binding site of gp130 (site 22) involves part of helix A, with an orientation that faces away from the CNTFRα site. The binding site of LIFR (site 3) is located within the D1 structural motif and involves residues of C-terminal portion of helix A and the N-terminal of A/B loop. It appears that the first 20 or so amino acid residues, as well as the last 15 residues at the C-terminus are not involved in receptor binding and could be used for the introduction of PEG moieties or other modifications. We identified the Cys 17 residue, which is not required for biological activity, as the suitable amino acid for conjugation with PEG. An additional advantage of using this residue for conjugation is that a free Cys can have a detrimental effect on long term stability of the protein owing to the formation of covalent aggregates, as in the similar cytokine G-CSF [27]. On the other hand, this Cys is not easily accessible for modification under the native protein conformation, likely due to burying inside a hydrophobic pocket as in the case of G-CSF [28]. In fact, in our hand Cys-17 PEGylation of DH-CNTF in PBS showed very low degrees of conversion. We already demonstrated with G-CSF that a tuned reversible denaturation of the protein might be a solution for temporally exposing the free Cys, thus allowing the coupling with PEG, followed by restoration of the native protein conformation. PEG-MAL 20 kDa was conjugated to DH-CNTF with an efficiency of 63% and the mono-PEGylated form was purified by RP-HPLC. The PEGylated form showed a single peak in analytical RP-HPLC (S1 File), as well as a single band in SDS-PAGE (Fig 2) of the apparent molecular weight of 60–80 kDa. The higher apparent MW of PEG-DH-CNTF, exceeding the expected MW, is attributable to the large hydrodynamic volume of the PEG moiety, leading to overestimation of real MWs of conjugates as known from the literature [29, 30], as demonstrated by the mass spectrometry spectra of DH-CNTF and PEG-DH-CNTF (S1 File).

**Fig 3:**
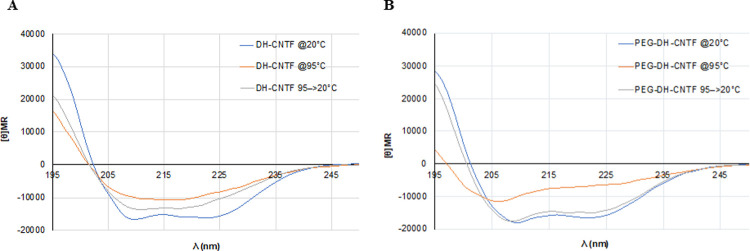
CD analysis of DH-CNTF and PEG-DH-CNTF at different temperatures. CD spectra were recorded at 20°C, 95°C and after cooling the samples from 95°C to 20°C. Samples were solubilized in water.

The secondary structure of the protein was not affected by PEGylation as shown by CD investigation (Fig 3). Interestingly, PEGylation stabilized the secondary structure of the protein during melting and favored the refolding when the heated sample of PEG-DH-CNTF was cooled from 95°C back to 20°C (Fig 3, panel B), as already observed for other PEGylated proteins [30]. The native protein under the same conditions did not fully restore the secondary structure (Fig 3 panel A).

### Identification of PEGylation site of PEG-DH-CNTF

**Fig 4:**
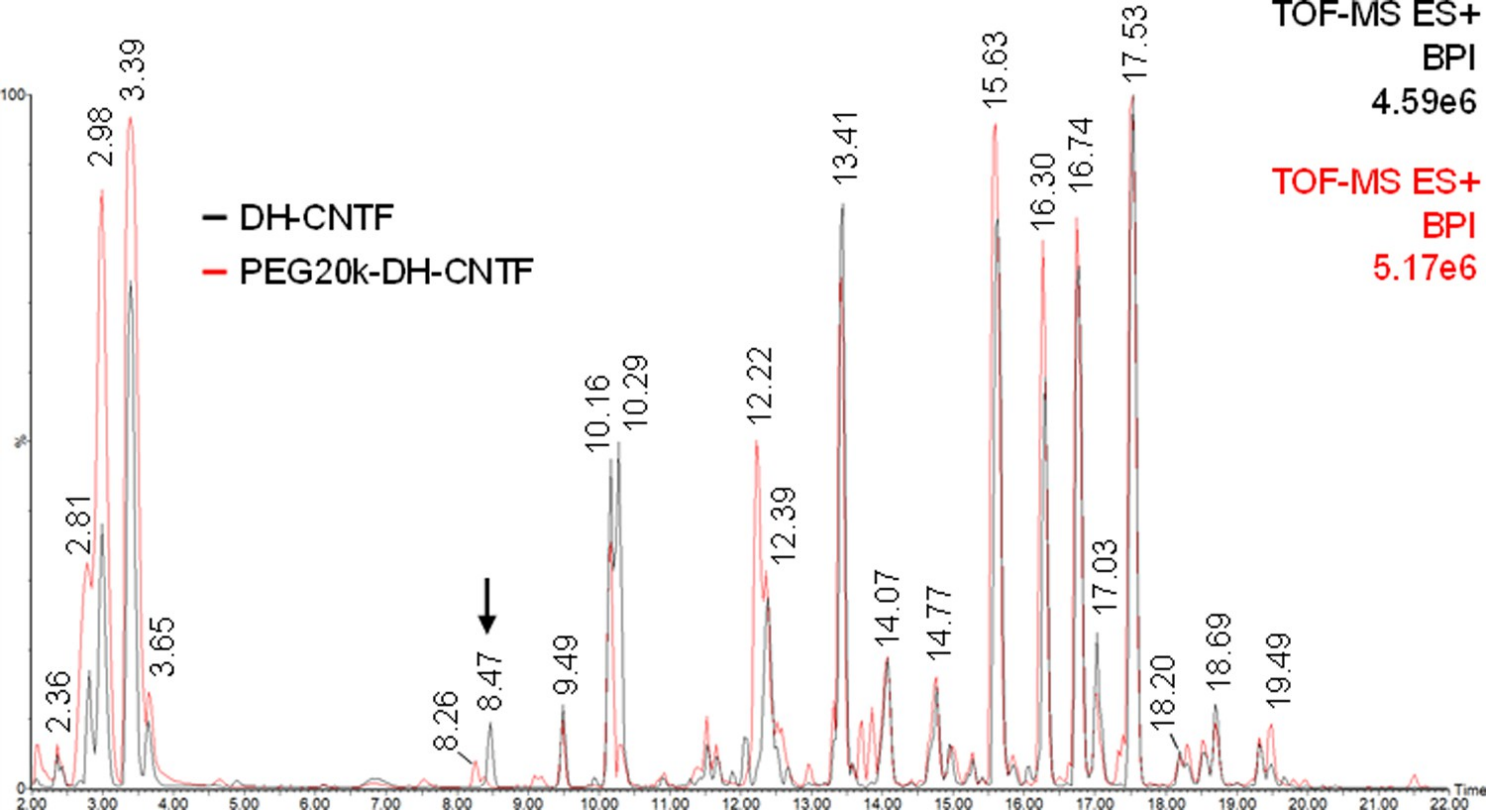
BPI chromatograms of LC-MS^E^ analysis of DH-CNTF (black) and PEG20k-DH-CNTF (red) upon digestion with trypsin. The peak labelled with an arrow in the figure is the Cys17-containing peptide F3 [15–19].

To experimentally identify the site of polymer conjugation, trypsin digestion of PEG-DH-CNTF and mass spectrometry analysis were performed. DH-CNTF was used as reference and treated under the same conditions. The LC-MS^E^ raw data were processed with BiopharmaLynx. A protein sequence coverage of 72% and 78% was found for DH-CNTF and PEG-DH-CNTF, respectively, and the identity of most of the found peptides was confirmed by b/y fragment ions (S1 File). Almost all the native DH-CNTF tryptic fragments were identified, except for the cysteine-lacking fragments F12 [90–133] and F22 [190–200], anyhow not relevant for the analysis. In the overlapped BPI chromatograms, extracted from MassLynx, each peak was matched to a peptide fragment and the profile of DH-CNTF digestion was compared to that of the PEGylated protein (Fig 4). The main difference between the two samples lies in the peak eluted at 8.47 min (calcd. [M] 649.2854 Da, found [M+H]^+^ 650.2908) that corresponds to the fragment F3 [15–19] containing Cys17. Since this peak was only present in DH-CNTF peptide profile and was detected with low intensity in the digested mixture of PEG-DH-CNTF, we may infer that the site of modification with PEG is the Cys17 residue. In fact, after PEGylation, the fragment could not be detected by QTof mass analysis.

### PEGylation of Cys17 did not alter the biological activity of DH-CNTF superagonist

**Fig 5:**
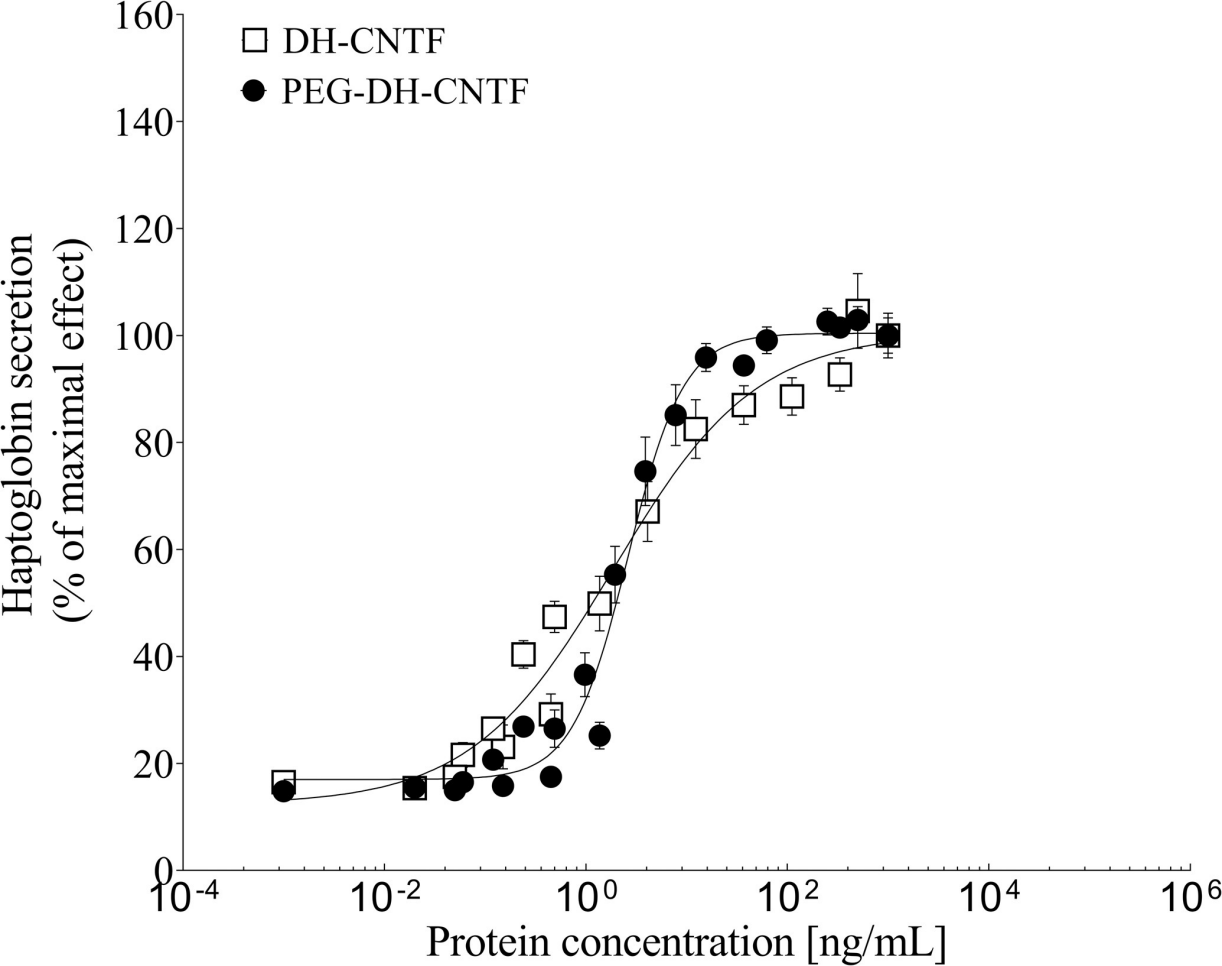
Biological activity of DH-CNTF and PEG-DH-CNTF in HepG2 cells. Results were expressed as a percentage of the maximal DH-CNTF-induced response and represent the mean ± S.E. from duplicate determinations. Data were from representative experiment repeated several times with comparable results.

The introduction of a PEG moiety had no effect on DH-CNTF potency, and the conjugate maintained the full potency, as reported in Fig 5. PEG-DH-CNTF was equipotent with DH-CNTF (EC_50_ value of 1.8 ± 0.2 ng/mL) in stimulating haptoglobin production in HepG2 cells with EC_50_ value of 2.5 ± 0.2 ng/mL.

Differently, previous attempts of site-specific attachment of different-sized PEG-maleimide or transferrin residue to wild-type CNTF indicated losses of activity in a survival assay of about 50–60% [19].

### Thioether specific PEGylation greatly prolonged serum half-life of DH-CNTF

In order to assess the expected gain in plasma stability by PEGylation, both proteins were administered via IV injection at 0.3 mg/kg in both mouse and rat. The DH-CNTF concentration in plasma was determined via the haptoglobin secretion assay, using plasma dilutions and standard curves of both DH-CNTF and PEGylated DH-CNTF.

**Fig 6:**
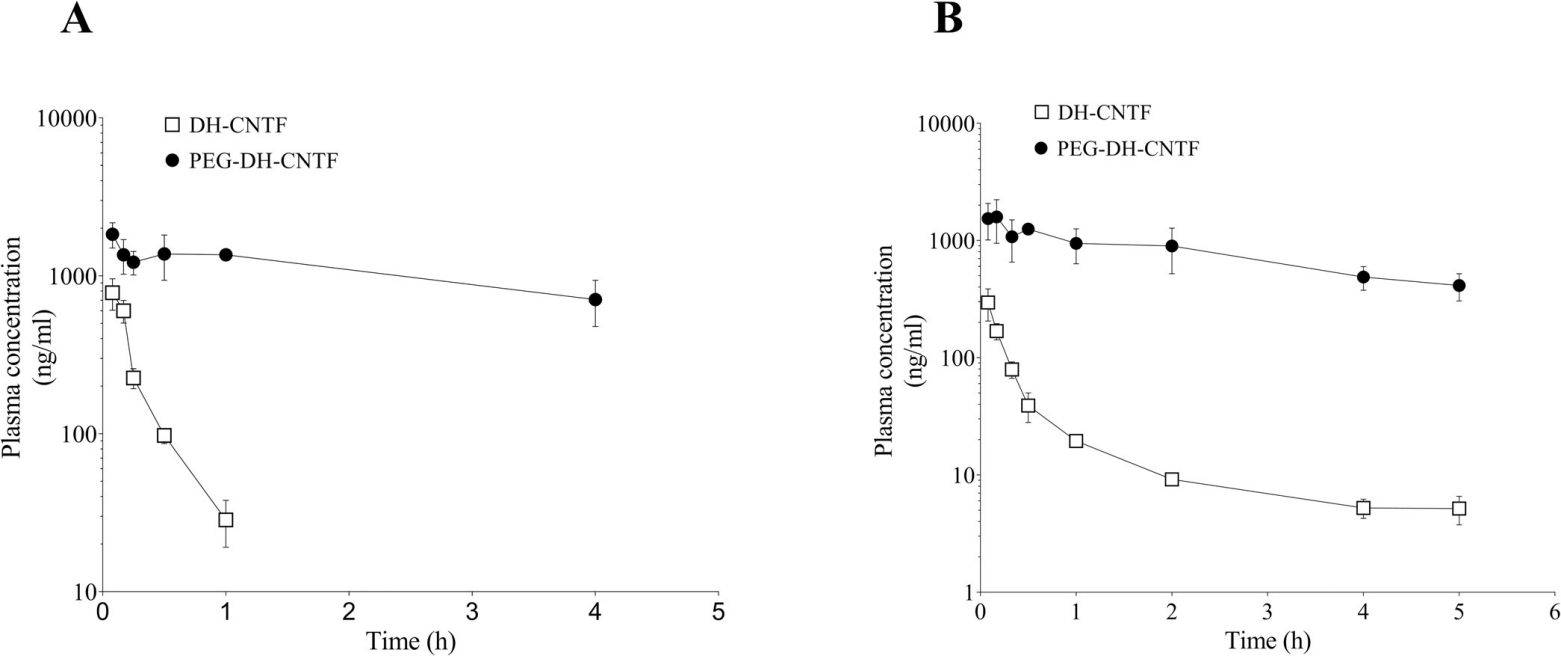
Plasma-time profiles in mouse (A) and rat (B) after IV injection of DH-CNTF and PEG-DH-CNTF. Values were the mean ± S.D. of duplicate determination of plasma obtained at different time points from 3 animals per group.

PEGylation resulted in a more than 10-fold increase in plasma half-life, from about 15 min to nearly 3 h, in both mouse and rat. A significant reduction of plasma clearance was also observed, while the volume of distribution was unchanged (Fig 6). The reduction of plasma clearance due to PEGylation could allow a reduction of the dose size and dosing frequency of PEG-DH-CNTF for reaching the desired effects *in vivo*, with a substantial increase of patient compliance and therapeutic outcomes.

**Fig 7:**
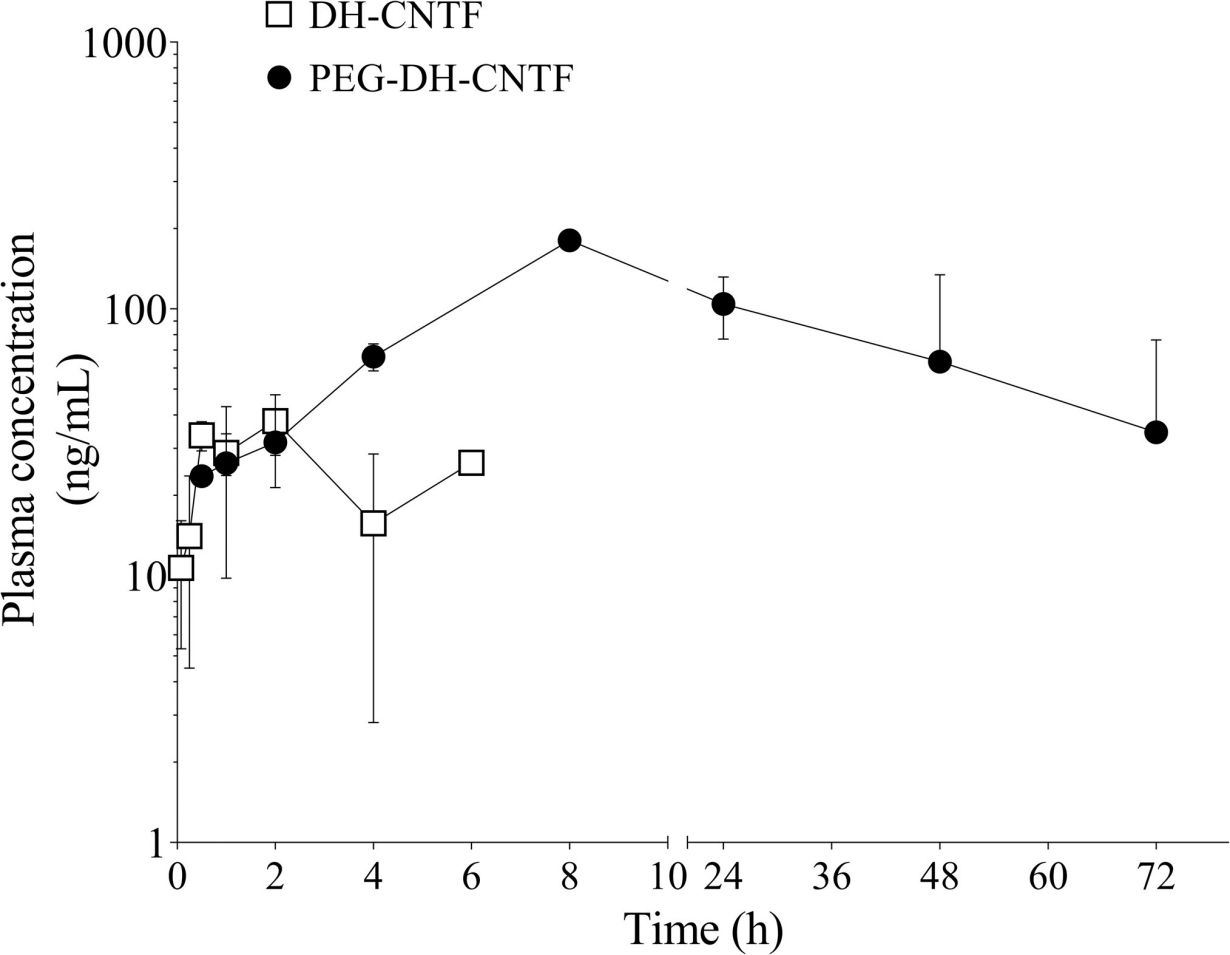
Plasma-time profiles concentration in mouse after SC injection of DH-CNTF and PEG-DH-CNTF.

PK behavior was also analyzed after sub-cutaneous injection of 1 mg/mL of DH-CNTF or PEG-DH-CNTF in mice. Fig 7 shows that PEG-DH-CNTF reached a higher C_max_ (180.8 ng/mL at 8 h) than DH-CNTF (37.9 ng/mL at 2 h), demonstrating that the extended pharmacokinetic characteristics were maintained after sub-cutaneous administration, with an overall bioavailability of about 30%. PK parameters both in mice and rats were reported in Table 2.

**Table 2:** Summary of pharmacokinetic data.

	Route	Specie	Dose [mg/kg]	T_1/2_ (min)	Clearance (mL/min/g)	Vd (L/kg)	C_max_ [ng/mL]	T_max_ (h)	AUC (ng*h/mL)	F (%)
**DH-CNTF**	IV	rat	0.3	30 ± 13	44 ± 9	1.9 ± 0.90	-	-	119 ± 27	-
	IV	mouse	0.3	15 ± 3	20 ± 2	0.5 ± 0.1	-	-	251 ± 25	-
	SC	mouse	1	54 ± 6	111 ± 13	8.7 ± 1.6	40 ± 6	1.5 ± 0.9	151 ± 17	18
**PEG-DH-CNTF**	IV	rat	0.3	156 ± 50	1.1 ± 0.3	0.3 ± 0.1	-	-	4783 ± 1603	-
	IV	mouse	0.3	185 ± 70	0.7 ± 0.2	0.17 ± 0.02	-	-	7843 ± 2867	-
	SC	mouse	1	1403 ± 821	3.4 ± 2.0	5.4 ± 2.3	126 ± 63	24 ± 0	7484 ± 6626	29

T_1/2_ = Half-life; Clearance = Apparent total body clearance of the drug from plasma; Vd = Apparent volume of distribution; C_max_ = Maximum peak plasma drug concentration; T_max_ = Time to reach maximum peak plasma concentration following drug administration; AUC = Area under the curve; F = Bioavailability

### Efficacy of PEGylated-DH-CNTF on body weight and food intake of Diet-Induced Obesity (DIO) model

**Fig 8:**
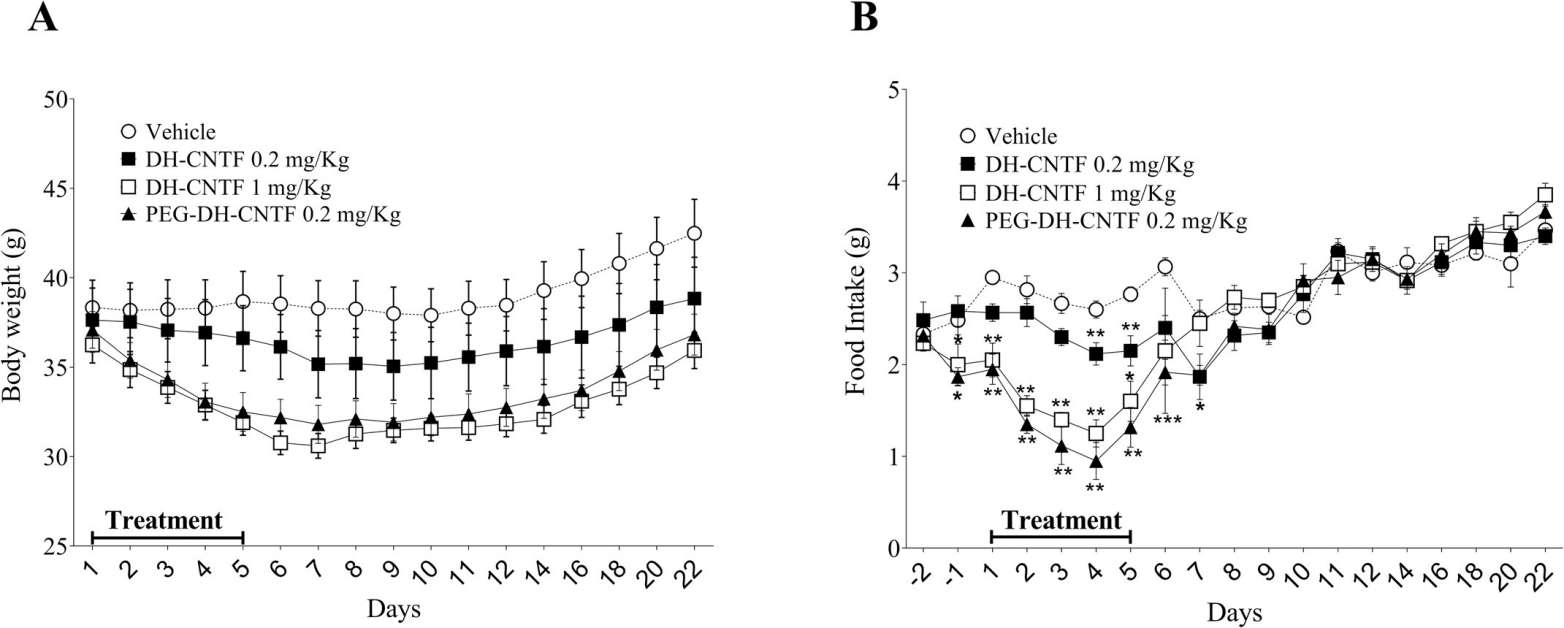
Effect of short-term administration of DH-CNTF and PEG-DH-CNTF in DIO mice. A) Body weight effect of 0.2 mg/kg and 1 mg/kg DH-CNTF and PEG-DH-CNTF (0.2 mg CNTF equivalent/kg), after daily SC administration. Body weight is expressed as grams starting before the first treatment and represents the mean ± SEM from 6 animals per group. Control group was treated with vehicle (0.9% saline/0.2 mg/mL endotoxin-free BSA). The arrow indicates the duration of treatment, starting from day 1 to day 5. B) Daily food consumption. Food consumption was calculated as the difference from the quantity proposed the day before and the remaining quantity. Statistical analysis (two-way ANOVA test: post-hoc Tukey’s Multiple Comparison Test) was performed for each group versus vehicle-treated group (*P < 0.05; **P < 0.01, ***P < 0.001).

Given the confirmed *in vitro* activity and the good pharmacokinetic profile of PEG-DH-CNTF, the next step was the evaluation of efficacy in an *in vivo* DIO model. DIO mice were made obese through feeding a high fat diet. DIO is a more human-like physiological model than the ob/ob or db/db mice models, based on the genetic defect of the leptin or its receptor, respectively [31]. In addition, mice placed on high fat diet display phenotypes similar to pre-diabetic humans and patients with metabolic disease, including increased weight gain and elevated blood glucose and insulin. As reported in Fig 8, PEG-DH-CNTF at 0.2 mg/kg was more potent than the non-PEGylated form at the same dosage in inducing weight loss after 5 days of treatment (-13% vs -3% from the starting weight). In fact, the PEGylation conferred a roughly 5 times higher efficacy to DH-CNTF, since almost the same weight reduction was obtained with 1 mg/kg of the non-PEGylated protein (-15%) or 0.2 mg/kg of PEG-DH-CNTF. However, at the end of the observation, 12 days after the last treatment, none of the treated groups had reached the weight of the control group, indicating that even a short-time treatment may have medium lasting effect. Decrease of about 60% of food intake in PEG-DH-CNTF-treated mice after 5 days of treatment was observed. Moreover, PEG-DH-CNTF treated mice, showed a more prolonged response than mice treated with 1 mg/kg DH-CNTF. Interestingly, the weight loss effect was protracted for additional two days after discontinuation of the treatment, while food intake started to increase one day before end of treatment. Consistent with previous observations [32], this effect could be due to the action of CNTF on energy expenditure and metabolic rate.

In this study the DH-CNTF treatment with SC injection of 1 mg/kg results in a slightly lower weight loss than previously reported for intraperitoneal treatment with 0.25 mg/kg [8]. This discrepancy likely results from the different route of DH-CNTF administration, with the IP route having higher bioavailability and/or more sustained release. In support of this hypothesis, twice weekly SC administration of wt CNTF at 1 mg/kg in another study [19] was unable to induce weight loss.

### Anti-diabetic effect of PEGylated DH-CNTF

**Fig 9:**
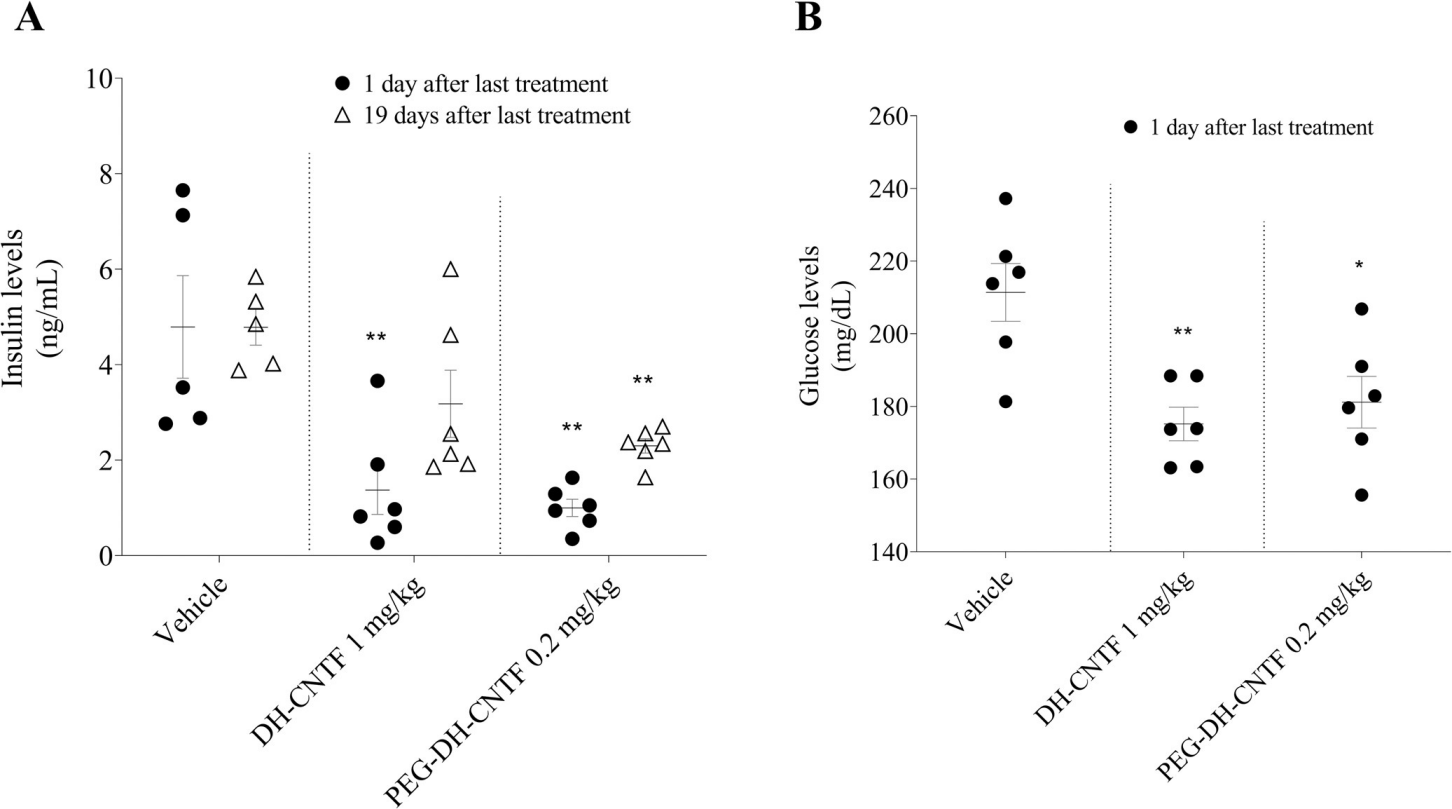
Effect on insulin and glucose in animal used for the weight loss. Statistical analysis (ANOVA test: post-hoc Dunnett’s Multiple Comparison Test) was performed for each group versus vehicle-treated group (*P < 0.05; **P < 0.01).

**Fig 10:**
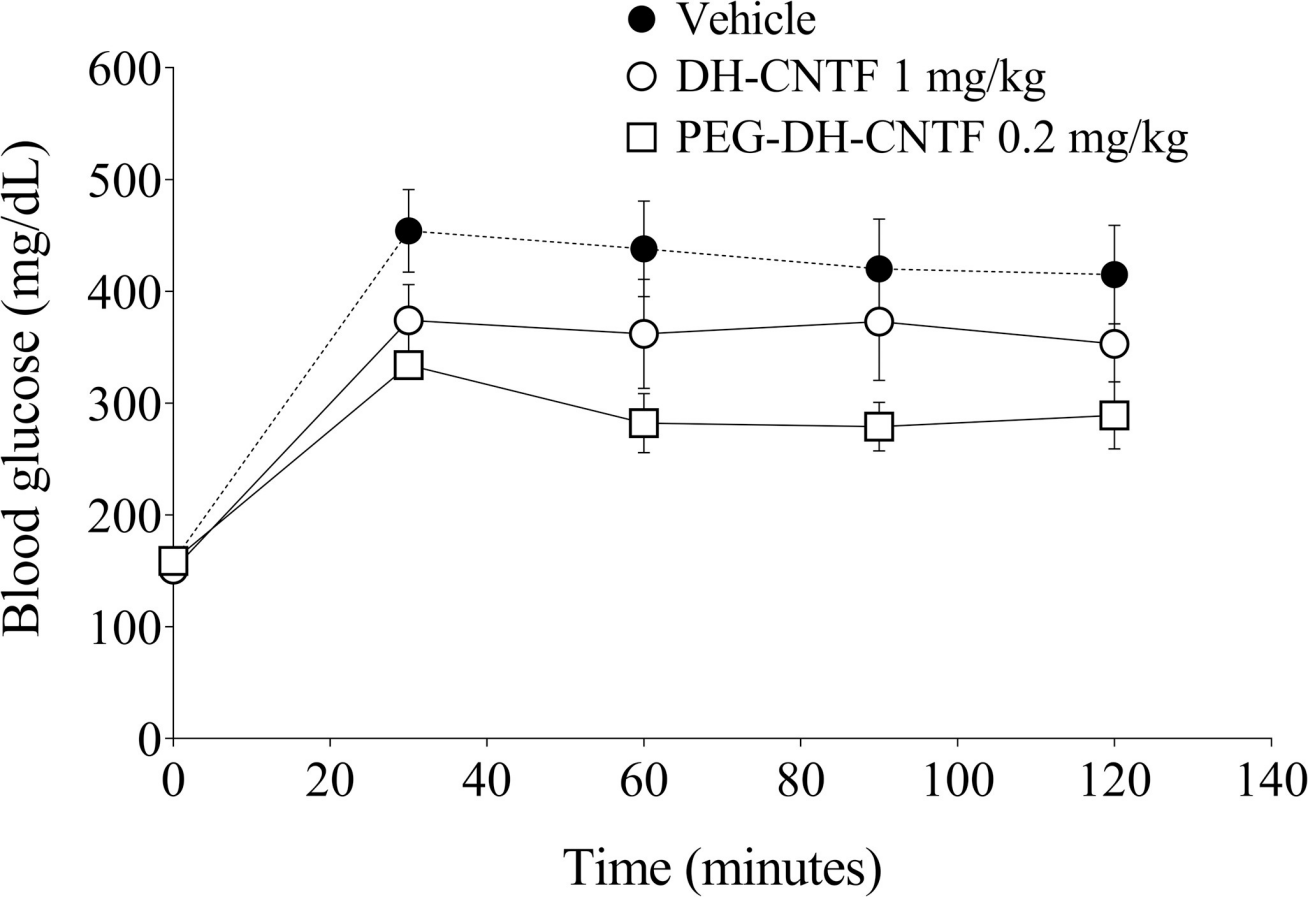
Intraperitoneal glucose tolerance test (IPGTT). Glucose levels, in DIO ♂ mice after IP administration of 1.5 mg/kg of glucose, were measured at 30 minutes intervals until 2 h.

DH-CNTF has been reported to possess anti-diabetic effect by reducing either the glycemia in db/db mice model or hyperinsulinemia in ob/ob mice [8]. Plasma insulin and glucose concentrations were significantly reduced in DIO mice treated with DH-CNTF and PEG-DH-CNTF at 1 mg/kg and 0.2 mg/kg, respectively (Fig 9 and Table 3). These data indicated that PEG-DH-CNTF treatment had a favorable effect not only on weight but also on the metabolic profile of obese mice. To further explore the effects of PEG-DH-CNTF on obesity-related factors, we carried out intraperitoneal glucose tolerance test (ipGTT) in DIO mice. ipGTT directly assesses glucose tolerance without being confounded by the possible alteration of incretin function. DIO mice exhibited fasting high glucose levels (159 ± 11 mg/dL, N = 6), that peaked at 454 mg/dL, 30 minutes after the intraperitoneal bolus glucose injection, indicating impaired glucose tolerance characteristic of this strain [33] As shown in Fig 10 and Table 4, PEGylated DH-CNTF was able to significantly decrease the glycemic peak with a dose 5-fold lower than DH-CNTF (0.2 mg/kg versus 1 mg/kg, respectively). This result obtained with less protein was in line with the pharmacokinetic data showing a circulating level of about 80 ng/mL for PEG-DH-CNTF as compared to around 20 ng/mL for DH-CNTF, 6 h after SC injection of the same dose of 1 mg/kg (Fig 7). In vehicle-treated animals the peak at 30 minutes was 454 mg/dL versus 334 mg/dL of mice receiving PEG-DH-CNTF (^§^P < 0.05). The treatment improved both the profile of blood glucose (Fig 10) and the area under the curve, AUC (Table 5).

**Table 3:** Effect of DH-CNTF and PEGylated protein administration for 5 days on circulating insulin and blood glucose concentrations in DIO mice used for the weight loss and food intake study.

Groups	Insulin [ng/mL]		Glucose [mg/dL]
	1 day after last treatment	19 days after last treatment	1 day after last treatment
**Vehicle**	4.8 ± 1.1	4.8 ± 0.4	211.4 ± 8.0
**DH-CNTF 1 mg/kg**	1.4 ± 0.5**	3.2 ± 0.7	175.1 ± 4.6**
**PEG-DH-CNTF 0.2 mg/kg**	1.0 ± 0.2**	2.3 ± 0.2**	181.1 ± 7.1*

Data were presented as means ± S.E. from groups composed of 5 to 6 mice. Data were obtained 1 day after the last treatment or in the post-treatment period (19 days after the last treatment). Statistical analysis (ANOVA test: post-hoc Dunnett’s Multiple Comparison Test) was performed for each group versus vehicle-treated group (*P < 0.05; **P < 0.01)

**Table 4:** IPGTT data in DIO mice. Starting Blood glucose levels in DIO mice were determined after 6 h fasting.

Groups	Time (minutes)				
	Pre-dose	30	60	90	120
**Vehicle**	159 ± 11	454 ± 37^§^	438 ± 43	420 ± 45	415 ± 44
**DH-CNTF 1 mg/kg**	151 ± 9	374 ± 32	362 ± 49	373 ± 53	353 ± 52
**PEG-DH-CNTF 0.2 mg/kg**	159 ± 3	334 ± 12^§^	282 ± 26	279 ± 22	289 ± 30

Data were presented as mean ± S.E. from groups composed of 5 to 6 mice. (^§^P < 0.05, T-test was performed using C_max_ values, at 30 minutes).

**Table 5:** Area under the curve (AUC) and C_max_ in glucose tolerance test.

Groups	AUC_0→2h_ (mg/dL*h)	C_max_ [mg/dL]
**Vehicle**	799 ± 69	454 ± 37
**DH-CNTF 1 mg/kg**	680 ± 80	374 ± 32
**PEG-DH-CNTF 0.2 mg/kg**	560 ± 30*	334 ± 12*

Data were presented as mean ± S.E. from groups composed of 5 to 6 mice. Statistical analysis (ANOVA test: post-hoc Dunnett’s Multiple Comparison Test) was performed for each DIO mice’s group versus vehicle-treated group (*P < 0.05)

## Conclusions

In the present study, we showed that the site selective conjugation of PEG to DH-CNTF at Cys17 allowed a significant improvement of its pharmacokinetic profile. Furthermore, PEG-DH-CNTF retained the protein activity, in fact, it decreased body weight and improved the metabolic profile of diet-induced obese mice even at a lower dose compared to the native protein. This demonstrates that a thoroughly designed conjugation strategy can yield a homogenous conjugate with improved *in vivo* performances for a better therapeutic exploitation.

The exact molecular mechanism by which PEG-CNTF exerts its anti-obesity and antidiabetic effect is not currently known. CNTF can bypass leptin resistance in obese animals, including leptin receptor-deficient mice [8, 10, 34], indicating that its action is not mediated *via* leptin. It has been suggested that the effect of CNTF on weight loss and insulin resistance may be mediated, in part, through alterations of secretion- and expression patterns of adiponectin and its receptors. Whether PEGylated DH-CNTF may affect the expression of adiponectin, adiponectin receptors or other adipokines in adipose and other tissues remains to be explored in future studies.

## Supporting information

S1 File (PDF)

S1 Raw image (PDF)
