# Supplementary material for: Efficacy of PEGylated ciliary neurotrophic factor superagonist variant in diet-induced obesity mice
Source: PLoS One. 2022 Mar 22;17(3):e0265749. doi: 10.1371/journal.pone.0265749 (PMC8939829; doi:10.1371/journal.pone.0265749)
Supplement: S1 File — (PDF) [file pone.0265749.s001.pdf]

Supplementary Material

Efficacy of PEGylated Ciliary Neurotrophic Factor super-agonist variant in diet-induced obesity mice

Maria Rosaria Battista[1], Antonella Grigoletto[2], Tommaso Tedeschini[2], Antonella Cellucci[1], Fabrizio Colaceci[1], Ralph Laufer[3], Gianfranco Pasut[2*] and Annalise Di Marco[1*]

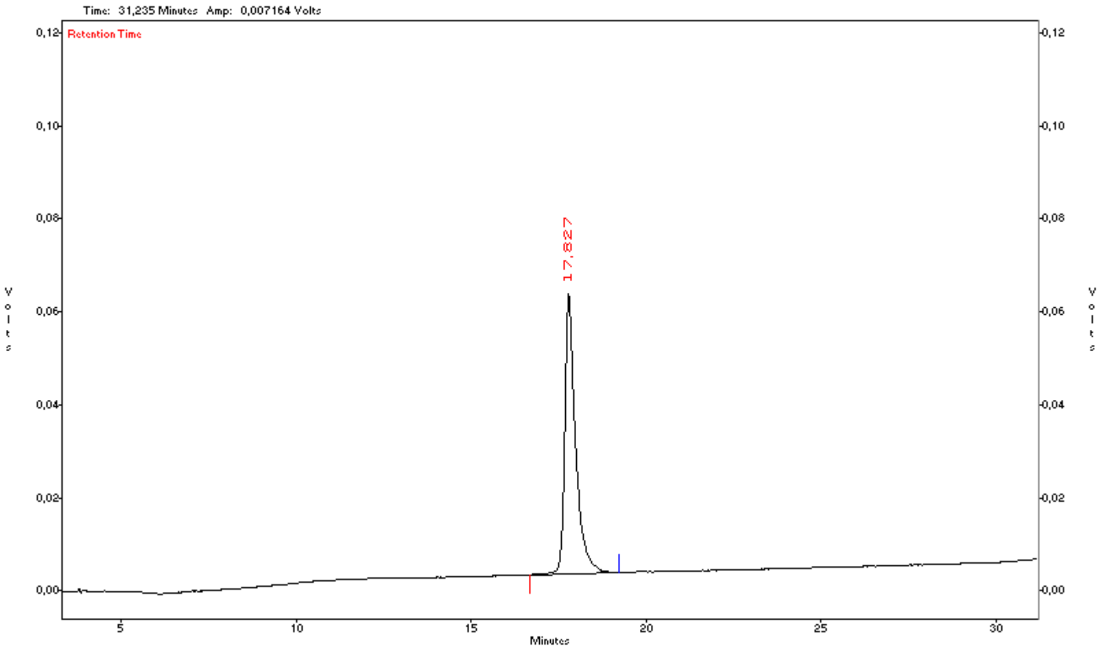

Fig S1. RP-HPLC chromatogram of DH-CNTF.
C18 column (Phenomenex Jupiter 250 × 4.6mm; 5µm; 300Å) eluted with a gradient of water and acetonitrile, both containing TFA 0.1% (v/v), gradient: 0'-5%, 5'-40%, 25'-80%, 27'-90%, 30'-5% B, flowrate of 1 mL/min, recording signal at 280 nm.

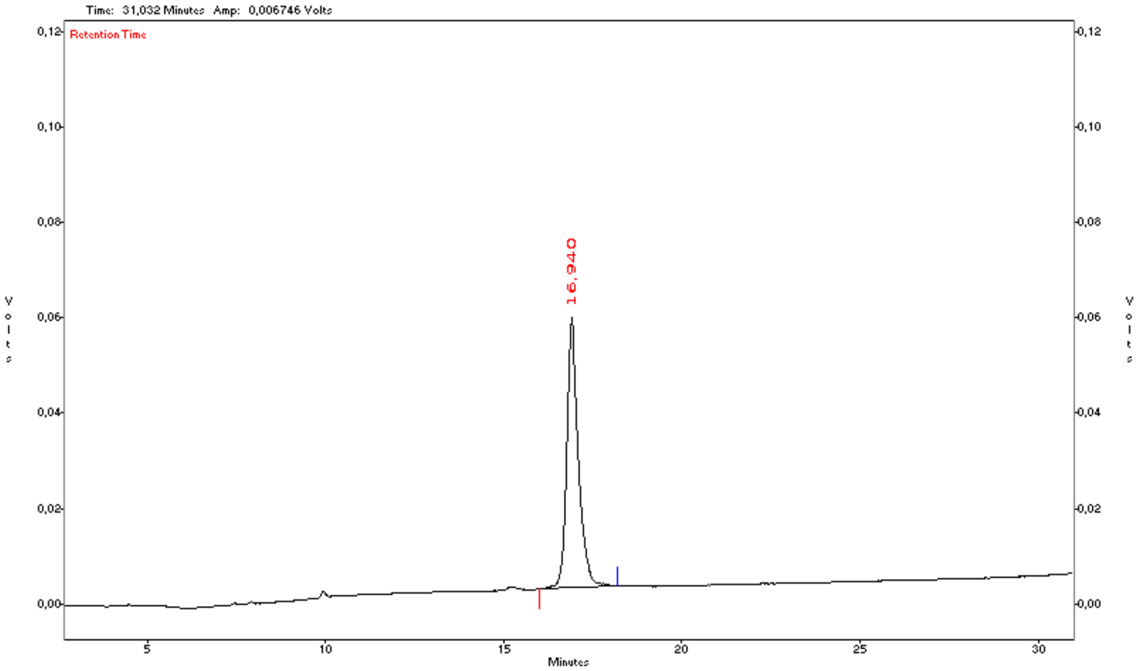

Fig S2. RP-HPLC chromatogram of PEG-DH-CNTF.
C18 column (Phenomenex Jupiter 250 × 4.6mm; 5µm; 300Å) eluted with a gradient of water and acetonitrile, both containing TFA 0.1% (v/v), gradient: 0'-5%, 5'-40%, 25'-80%, 27'-90%, 30'-5% B, flow rate of 1 mL/min, recording signal at 280 nm

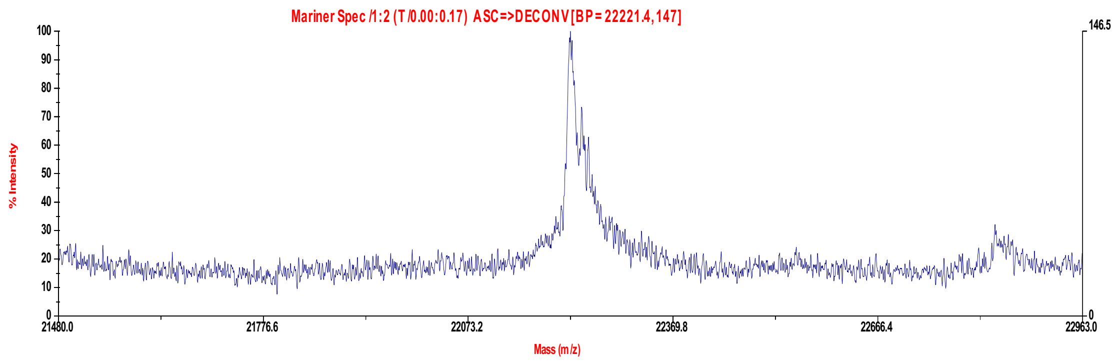

Fig S3. MALDI mass spectra of DH-CNTF.

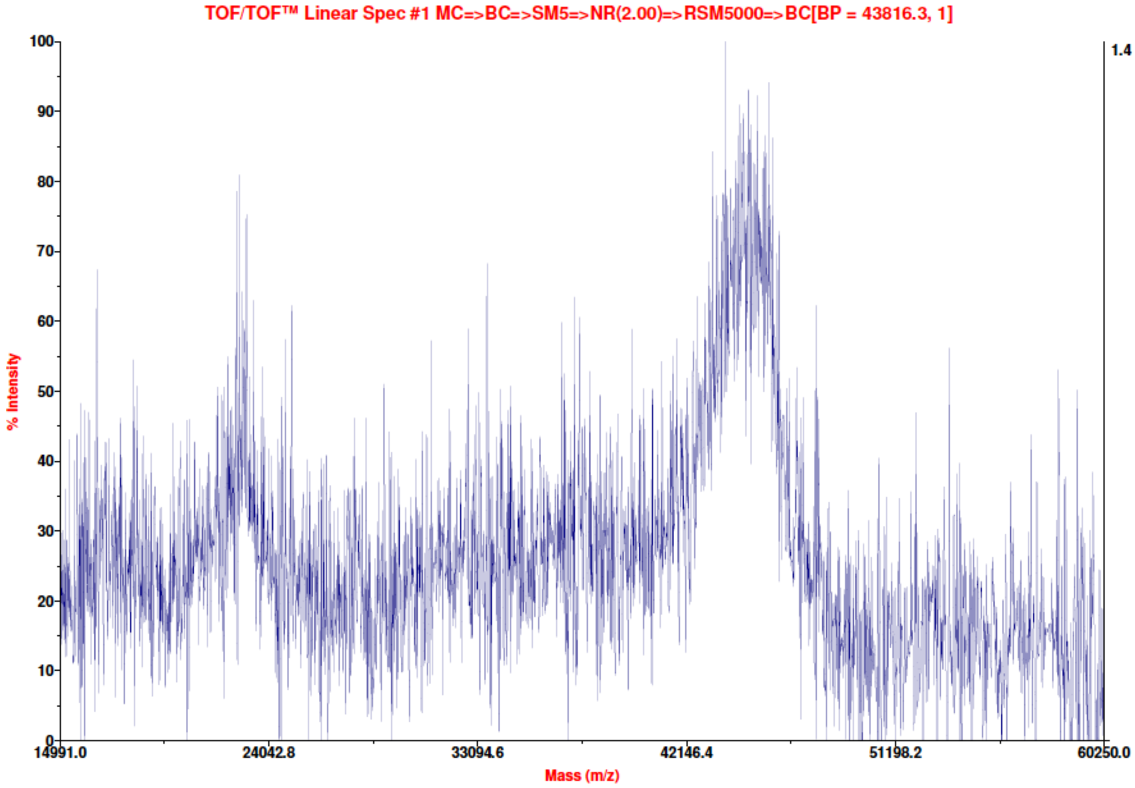

Fig S4. MALDI mass spectra of PEG-DH-CNTF.

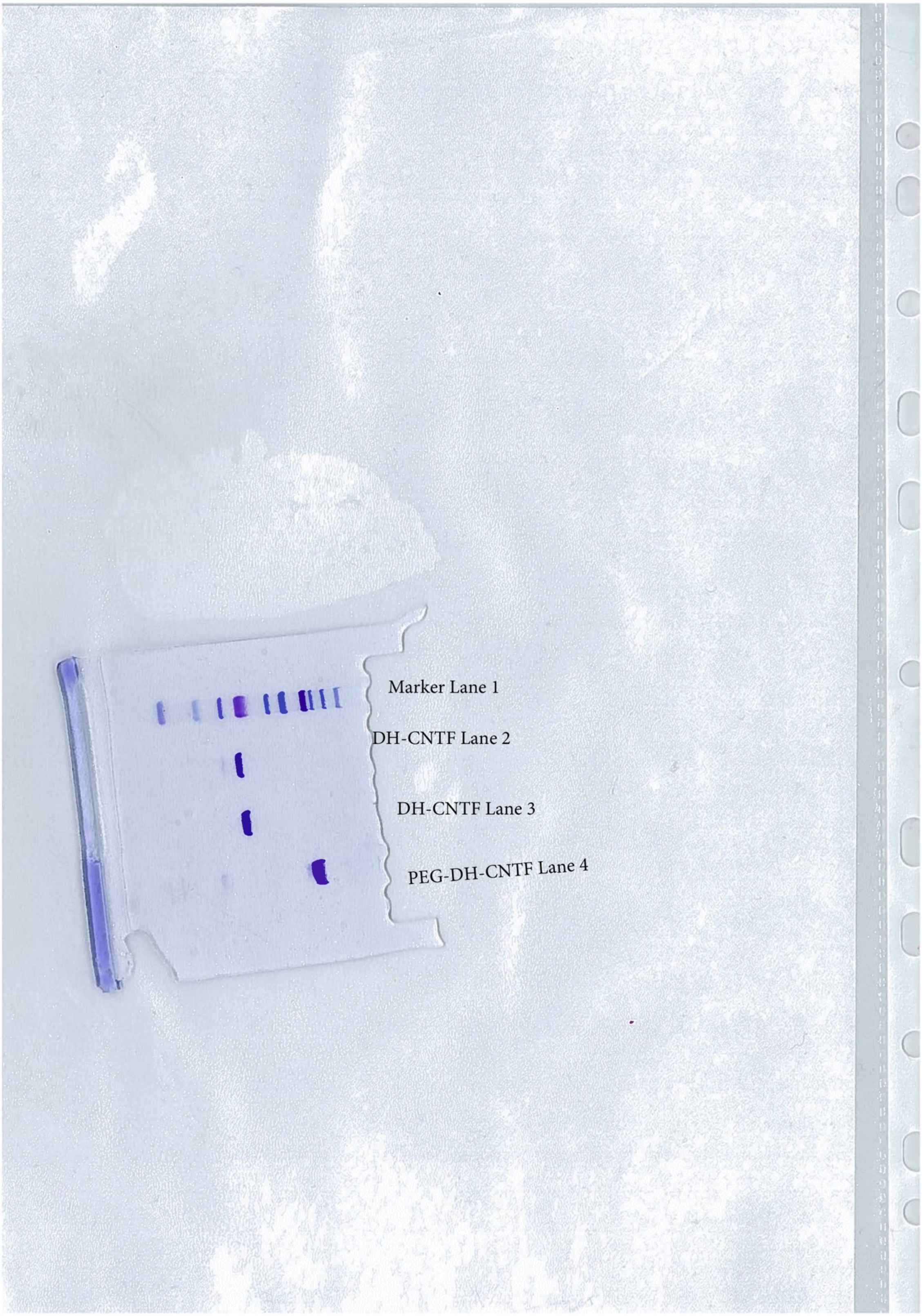

Fig. S5. Raw SDS-PAGE gel image

Table S1. Blood sampling.

Route	Group #	Time points	# of mice/group	# Total mice
IV	1	Pre-dose	3	12
	2	0.08 h – 0.5 h	3	
	3	0.17 h – 1 h	3	
	4	0.50 h – 4 h	3	
SC	1	Pre-dose	3	15
	2	0.08 h – 2 h – 24 h	3	
	3	0.25 h – 4 h – 48 h	3	
	4	0.50 h – 6 h – 72 h	3	
	5	1 h – 8 h	3	

Table S2. Tryptic peptides of native DH-CNTF obtained upon digestion with trypsin. Molecular masses were determined by LC-MS^E.

Sequence	Fragment	Start	End	RT (Min)	Calculated Peptide Mass	Measured Peptide Mass	b/y possible	b/y found	Intensity (Counts)
MAFTEHSPLTPHR	F1	1	13	29.17	1522.7351	1522.7574	24	0	4532
MAFTEHSPLTPHRR	F1-2	1	14	29.7	1678.8362	1678.8221	26	1	1307
DLCSR	F3	15	19	8.47	649.2854	649.2857	8	6	155813
SIWLAR	F4	20	25	16.3	744.4282	744.4291	10	9	1384399
SIWLARKIR	F4-6	20	28	16.9	1141.7084	1141.7015	16	1	2606
KIR	F5-6	26	28	2.36	415.2907	415.2898	4	2	72009
KIRSDLTALTESYVK	F5-7	26	40	15.8	1722.9515	1722.9503	28	12	207232
IRSDLTALTESYVK	F6-7	27	40	17.1	1594.8566	1594.8503	26	11	25704
SDLTALTESYVK	F7	29	40	17.53	1325.6714	1325.6732	22	21	4696081
HQGLNK	F8	41	46	3.65	695.3715	695.3715	10	9	187552
NINLDSADGMPVAST DQWSELTEAER	F9	47	72	19.49	2848.2820	2848.2747	50	41	507229
NINLDSADG**M**PVAST DQWSELTEAER	F9 (ox)	47	72	18.2	2864.2771	2864.2715	50	38	720667
LQENLQAYR	F10	73	81	13.41	1133.5829	1133.5865	16	15	2902668
TFHVLLAR	F11	82	89	15.63	955.5603	955.5672	14	13	2356594
IPR	F13	134	136	6.9	384.2485	384.2463	4	2	42171
NEADGMPINVGDGG LFEK	F14	137	154	18.69	1861.8516	1861.8480	34	29	1102907
NEADG**M**PINVGDGG LFEK	F14 (ox)	137	154	17.1	1877.8464	1877.8414	34	30	1275047
KLWGLK	F15-16	155	160	14.77	743.4694	743.4695	10	10	369821
LWGLK	F16	156	160	17.0	615.3744	615.3753	8	7	276574
VLQELDHWTVR	F17	161	171	16.8	1394.7306	1394.7352	20	17	2616776
SIHDLR	F18	172	177	10.16	739.3977	739.4006	10	9	766219
FISSHQTGIPAR	F19	178	189	12.3	1312.6887	1312.6879	22	11	111177
GSHYIANNK	F20	190	198	-	1002.4883	-			-
GSHYIANNKK	F20-21	190	199	-	1130.5833	-			-

Table S3. Tryptic peptides of PEG20k-DH-CNTF obtained upon digestion with trypsin. Molecular masses were determined by LC-MS^E.

Sequence	Fragment	Start	End	RT (Min)	Calculated Peptide Mass	Measured Peptide Mass	b/y possible	b/y found	Intensity (Counts)
MAFTEHSPLTPHR	F1	1	13	29.17	1522.7351	1522.7574	24	0	4532
MAFTEHSPLTPHRR	F1-2	1	14	29.7	1678.8362	1678.8221	26	1	1307
DLCSR	F3	15	19	8.4	649.2854	649.2827	8	2	6269
SIWLAR	F4	20	25	16.26	744.4282	744.4309	10	9	2786968
SIWLARKIR	F4-6	20	28	16.9	1141.7084	1141.7015	16	1	2606
KIR	F5-6	26	28	2.32	415.2907	415.2904	4	3	108419
KIRSDLTALTESYVK	F5-7	26	40	15.8	1722.9515	1722.9503	28	12	207232
IRSDLTALTESYVK	F6-7	27	40	17	1594.8566	1594.8558	26	18	300406
SDLTALTESYVK	F7	29	40	17.53	1325.6714	1325.6766	22	21	6737341
HQGLNK	F8	41	46	3.65	695.3715	695.3720	10	10	341185
NINLDSADGMPVASTDQWSELTEAER	F9	47	72	19.49	2848.2820	2848.2778	50	27	992557
NINLDSADG**M**PVASTDQWSELTEAER	F9 (ox)	47	72	18.3	2864.2771	2864.2700	50	37	755640
LQENLQAYR	F10	73	81	13.41	1133.5829	1133.5870	16	15	3242156
TFHVLLAR	F11	82	89	15.63	955.5603	955.5724	14	13	3120719
IPR	F13	134	136	6.8	384.2485	384.2467	4	2	42257
NEADGMPINVGDGGLFEK	F14	137	154	18.69	1861.8516	1861.8478	34	24	988059
NEADG**M**PINVGDGGLFEK	F14 (ox)	137	154	17.1	1877.8464	1877.8433	34	29	1154820
KLWGLK	F15-16	155	160	14.8	743.4694	743.4697	10	10	555059
LWGLK	F16	156	160	17.0	615.3744	615.3755	8	7	243719
VLQELDHWTVR	F17	161	171	16.8	1394.7306	1394.7374	20	17	3479875
SIHDLR	F18	172	177	10.2	739.3977	739.4006	10	9	771232
FISSHQTGIPAR	F19	178	189	12.2	1312.6887	1312.6910	22	18	2066994
GSHYIANNK	F20	190	198	8.3	1002.4883	1002.4874	16	12	102127
GSHYIANNKK	F20-21	190	199	6.4	1130.5833	1130.5778	18	2	31891
