# Supplementary figures and images for: Efficacy of PEGylated ciliary neurotrophic factor superagonist variant in diet-induced obesity mice
Source: PLoS One. 2022 Mar 22;17(3):e0265749. doi: 10.1371/journal.pone.0265749 (PMC8939829; doi:10.1371/journal.pone.0265749)

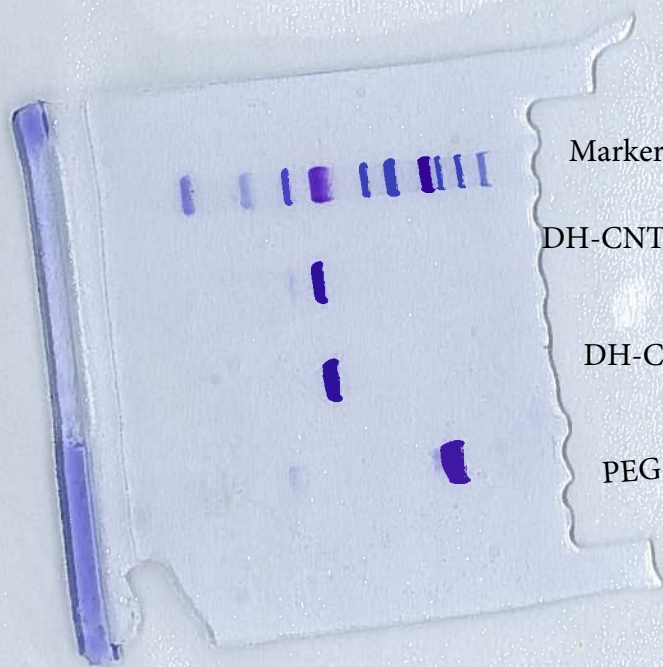

Marker Lane 1

DH-CNTF Lane 2

DH-CNTF Lane 3

PEG-DH-CNTF Lane 4

Supplement: S1 Raw image — (PDF) [file pone.0265749.s002.pdf]
